# First person – Eileen Lynch

**DOI:** 10.1242/dmm.041640

**Published:** 2019-08-16

## Abstract

First Person is a series of interviews with the first authors of a selection of papers published in Disease Models & Mechanisms (DMM), helping early-career researchers promote themselves alongside their papers. Eileen Lynch is first author on ‘C9ORF72-related cellular pathology in skeletal myocytes derived from ALS-patient induced pluripotent stem cells’, published in DMM. Eileen is a graduate research assistant/PhD candidate in the lab of Masatoshi Suzuki at University of Wisconsin-Madison, WI, USA, investigating *in vitro* disease modeling of neuromuscular diseases.


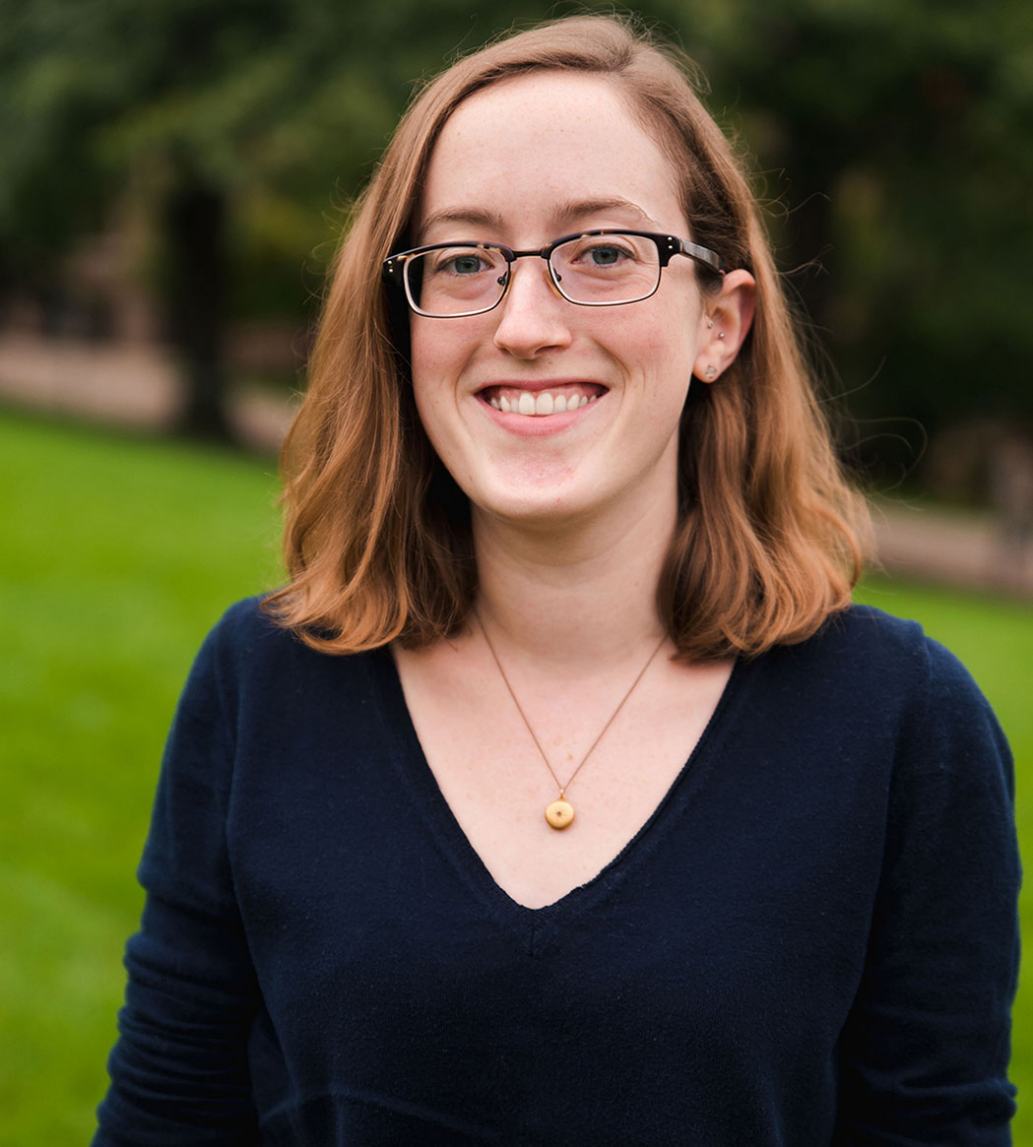


**Eileen Lynch**

**How would you explain the main findings of your paper to non-scientific family and friends?**

Amyotrophic lateral sclerosis, also known as ALS or Lou Gehrig's disease, is a fatal neuromuscular disease in which patients become gradually paralyzed and ultimately die from respiratory failure. The *C9ORF72* mutation was recently discovered to be the most common ALS-causing mutation, but the specific mechanisms by which this mutation influences the cells of ALS patients are still being determined. A major focus has been on how the mutation affects motor neurons, but very little is known about how it could be affecting skeletal muscle cells, or skeletal myocytes. Our work used induced pluripotent stem cells (iPSCs) from ALS patients with the *C9ORF72* mutation and differentiated them into skeletal muscle cells to characterize the mutation in this specific cell type. We found that the muscle cells prepared from patient-derived stem cells contained cellular abnormalities specific to the *C9ORF72* mutation that had previously mainly been characterized in motor neurons. In addition, the muscle cells had significant changes in the expression of genes related to mitochondrial function, a susceptibility to oxidative stress, and protein aggregation. These findings suggest a possible role of skeletal muscle in ALS disease progression.

**What are the potential implications of these results for your field of research?**

Our results support the hypothesis that ALS-patient-derived skeletal muscle cells experience pathological changes independent of motor neuron influence. Some of our next steps include comparing our iPSC-derived skeletal muscle pathology to actual patient muscle samples to determine at what stage in the disease process these changes are occurring. If skeletal muscle experiences pathological changes earlier than expected in the ALS disease progression then it could lead to novel biomarkers or therapeutic targets.

“These findings suggest a possible role of skeletal muscle in ALS disease progression.”

**What are the main advantages and drawbacks of the model system you have used as it relates to the disease you are investigating?**

iPSCs are a great resource for *in vitro* disease modeling because they can be generated from easily accessible adult cell types such as skin fibroblasts or blood cells. Once they have been reprogrammed to a pluripotent state they continue to proliferate indefinitely, generating a limitless pool of cells to work with. They also contain the genetic makeup of the patient that they came from, so they can be differentiated into a cell type of interest and then studied to see how that cell type is influenced by the disease-causing mutation. A drawback of our model system would be the differentiation efficiency, which results in a heterogeneous population of cells. This can make it difficult to quantify certain effects on skeletal myocytes independent of the influence of the other cell types in culture. This is why immunocytochemistry is important, to confirm that the mechanism is actually occurring specifically in the skeletal myocytes.

**What has surprised you the most while conducting your research?**

I was pleasantly surprised to find that characteristics of the *C9ORF72* mutation (such as repeat RNA foci and aggregation of repeating dipeptides) that have been well characterized in motor neurons are also present in skeletal myocytes. In particular, it was interesting to see such wide variation in the number of repeat RNA foci that were present in the nuclei of skeletal myocytes, even comparing nuclei within the same myocyte.

“I think that the most significant challenge for treating ALS patients is that around 90% of cases are considered sporadic or randomly acquired, and the other 10% are genetically inherited mutations, of which there are many.”

**Describe what you think is the most significant challenge impacting your research at this time and how will this be addressed over the next 10 years?**

I think that the most significant challenge for treating ALS patients is that around 90% of cases are considered sporadic or randomly acquired, and the other 10% are genetically inherited mutations, of which there are many. This may mean that treatment options will need to be individually tailored to the patient, rather than one specific treatment that can work for every patient. Genetic testing will be necessary to determine treatment options. Another challenge is the quick progression of ALS. By the time the disease is diagnosed, patients only live on average 3-5 years. Therefore, there is a critical need for biomarkers that can help to detect and diagnose the disease much earlier. This will allow treatment to begin sooner in order to have a better chance of prolonging the patient's lifespan.

**Figure:**
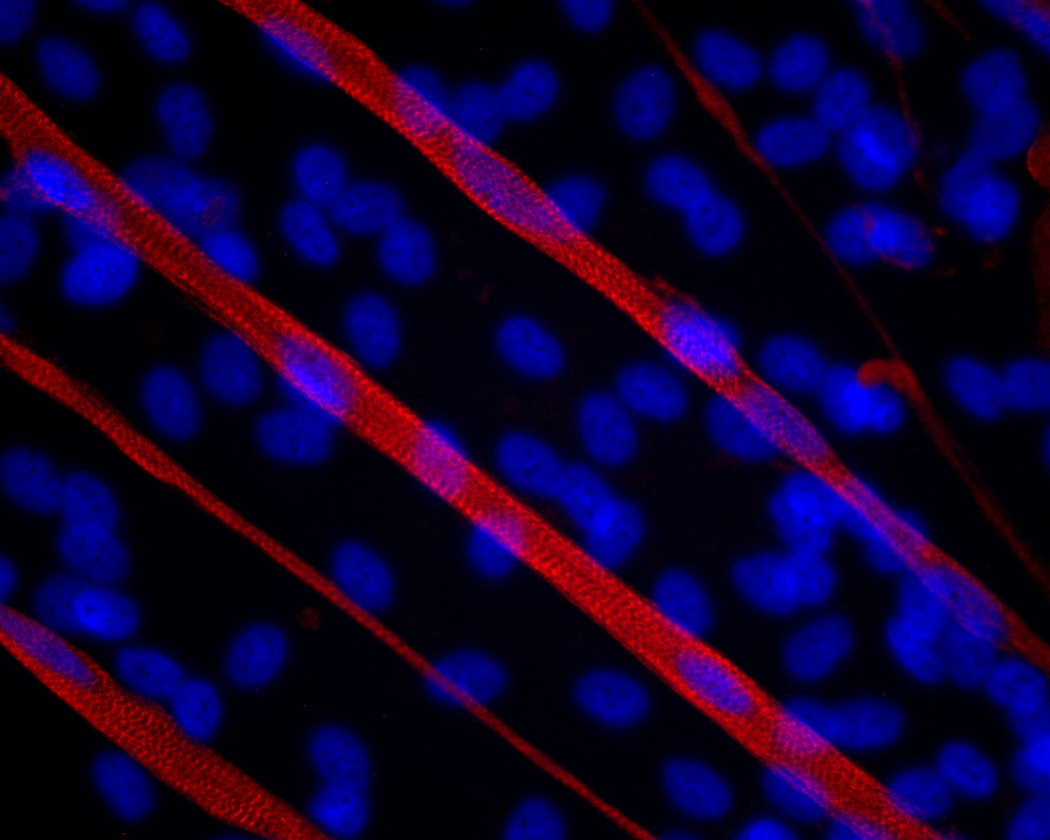
**Immunostaining for the sarcomeric protein titin shows nicely defined striations in iPSC-derived skeletal myocytes.**

**What changes do you think could improve the professional lives of early-career scientists?**

I think that better access to exploring career options is needed in many graduate programs. The individual development plans encouraged by the NIH are a step in the right direction, but universities should also offer programs to give students a better idea of the wide range of careers they can pursue with their degree. Internships can be difficult to squeeze into a demanding PhD timeline, but should be encouraged by graduate programs and PIs.

**What's next for you?**

For my additional projects I am interested in common pathogenic mechanisms that occur in the skeletal muscle of ALS patients across the various disease backgrounds, as well as *in vitro* co-culture models of motor neurons and skeletal myocytes in order to study what is happening at the neuromuscular junction of ALS patients. I hope to complete my PhD in the next year. I'd like to continue working in academia, so I am searching for a postdoctoral position in a lab where I could continue to study neuromuscular diseases and possibly move into tissue-engineering 3D models of skeletal muscle pathology.
